# Draft Genome Sequence of Beta-Hemolytic *Streptococcus iniae* KCTC 11634

**DOI:** 10.1128/genomeA.00897-13

**Published:** 2013-11-07

**Authors:** Hye Sung Choi, Mun Gyeong Kwon, Myoung Sug Kim, Myoung Ae Park, Dong-Wook Kim, Jin-Young Park, Ji-Sun Kim, Yun-Jeong Na, Min-Young Kim, Dae-Soo Kim, Sung-Hwa Chae, Jung Soo Seo

**Affiliations:** Pathology Division, National Fisheries Research and Development Institute (NFRDI), Busan, Republic of Koreaa; Human Derived Material Center, Korea Research Institute of Bioscience and Biotechnology (KRIBB), Daejeon, Republic of Koreab; Research Institute of GnCBIO Co., Ltd., Daejeon, Republic of Koreac

## Abstract

*Streptococcus iniae* is a beta-hemolytic, Gram-positive coccus, which affects a broad range of freshwater and marine fish species, causing substantial economic losses in the aquaculture industry worldwide. Thus, it is very important to derive a complete genome sequence of the bacterium to aid in the development of vaccines and methods for preventing fish streptococcosis and zoonotic infections in humans. Here, we present the draft genome sequence of *S. iniae* KCTC 11634 (1,955,615 bp, with a G+C content of 36.6%), which contains 1,868 putative coding sequences.

## GENOME ANNOUNCEMENT

*Streptococcus iniae* is a beta-hemolytic, Gram-positive coccus, which affects a broad range of freshwater and marine fish species, causing substantial economic losses in the aquaculture industry worldwide (1). Through originally isolated from the subcutaneous lesions of a captive Amazon freshwater dolphin, *Inia geoffrensis*, in 1976 (2), *S. iniae* is predominantly a fish pathogen with a broad host range of fresh and saltwater species, such as trout, tilapia, salmon, barramundi, yellowtail, flounder, and hybrid striped bass (1). Streptococcosis caused by *S. iniae* has increased mortality over a period of several weeks in the dominant aquaculture industry of *Paralichthys olivaceus* in South Korea. More importantly, humans can also become infected by *S. iniae*, and a number of human cases have been reported in North America and Asia (3, 4).

Genomic DNA was extracted from the cultured bacteria using the alkaline lysis method (5). We sequenced the genome of this species because it had not been sequenced at the time our sequencing project began, according to the Genomes OnLine Database (GOLD) (6).

We report the genome sequence of *S. iniae* KCTC 11634, obtained using a whole-genome shotgun strategy (7, 8) with a Roche 454 GS (FLX Titanium) pyrosequencing system (760,988 reads totaling ~199.9 Mb, for ~28.9-fold coverage of the genome). Pyrosequencing was processed using Roche’s software, according to the manufacturer’s instructions. All of the paired reads were assembled using the Newbler assembler 2.6 (454 Life Science), which generated 174 contigs (accession no. BANM01000001 to BANM01000174) (largest contig, ~170 kb) of >100 bp in size. The initial draft assembly contains 69 contigs in 8 scaffolds. The predicted proteins were annotated using Basic Local Alignment Search Tool (BLAST) (9) and the Rapid Annotations using Subsystems Technology (RAST) server (10). In addition, open reading frame (ORF) prediction was merged to use the CD-HIT software to search contigs against the Glimmer 3.02 modeling software package and Genemark version 2.5 (11), tRNAscan-SE 1.21 (12), RNAmmer 1.2 (13), and Clusters of Orthologous Groups (COG) (14) databases to annotate the gene descriptions.

The *S. iniae* draft genome includes 1,955,615 bp and is composed of 1,868 predicted coding sequences (CDSs), with a G+C content of 36.6%. The genome contains representatives of 307 subsystems, and we used this information to reconstruct the metabolic network (determined using the RAST server). A distinguishing subsystem feature was the absence of a gene corresponding to ABC transporter ATP-binding protein, DNA polymerase, DNA repair protein, membrane protein, repeat-containing cell surface protein precursor, transcriptional regulator. The CDSs annotated by the COG database were classified into 8 major categories (R, E, S, K, G, P, J, and L) from among the 46 COG groups. The enzymes identified included the phosphotransferase system (PTS) (EC 2.7.1.69), DNA polymerase III (EC 2.7.7.7), and RNA pseudouridylate synthase (EC 5.4.99.12). A more detailed analysis of this genome and a comparative analysis with other *S. iniae* genomes may identify other genes upon finalization of the genome.

### Nucleotide sequence accession numbers.

The draft genome sequence of *S. iniae* KCTC 11634 is available in GenBank under the accession no. BANM00000000 (DF266798 to DF266805).
